# Acute caffeine intervention and cognitive performance in populations aged 20 years and younger: a systematic review and meta-analysis

**DOI:** 10.3389/fnut.2026.1888658

**Published:** 2026-09-24

**Authors:** Jianfeng Niu, Haobai Li, Ziyi Wang, Lingxin Xu, Liangru Liu, Jinyu Liu, Jia Li, Yecheng Cao

**Affiliations:** 1 Sports Coaching College, Beijing Sport University, Beijing, China; 2 Physical Education Department, Beijing International Studies University, Beijing, China; 3 School of Physical Education, Guangxi University, Nanning, Guangxi, China; 4 School of physical education, Hunan University, Changsha, China; 5 Dalian Jiaotong University, Dalian, China; 6 Department of Sports Training, Hebei Sport University, Shijiazhuang, Hebei, China

**Keywords:** caffeine, cognitive function, dose–response, meta-analysis, young population

## Abstract

**Objectives:**

This systematic review and meta-analysis aimed to evaluate the acute effects of caffeine supplementation on cognitive performance in healthy populations aged 20 years and younger, and to explore potential moderators including dose, cognitive domain, population type, and outcome measure.

**Methods:**

Four databases (Web of Science, PubMed, MEDLINE, and Cochrane Library) were searched from inception to March 2026. Randomized controlled trials and randomized crossover trials assessing the effects of acute oral caffeine versus placebo on cognitive outcomes in individuals aged ≤20 years were included. A two-level meta-analytic model was used to calculate pooled standardized mean differences (SMD). Subgroup analyses and meta-regression were performed to explore potential methodological moderators influencing the overall effect.

**Results:**

Fourteen studies comprising 250 participants and 37 effect sizes were included. Caffeine demonstrated a small positive effect on cognitive task performance compared with placebo (SMD = 0.41, 95% CI 0.28–0.55; *p* < 0.01), with low heterogeneity (I^2^ = 10%). Meta-regression showed no significant moderating influences of age, acute supplementation dose, habitual daily intake, and acute-to-habitual dose ratio on caffeine-induced cognitive improvements. However, limited statistical power may restrict the interpretability of this result. Subgroup analyses showed that cognitive domain, outcome measure type, participant population, administration route, and sex did not significantly moderate caffeine’s cognitive effects. Notably, however, when comparing outcome measure types, the enhancement magnitude of caffeine on reaction time was greater than that on accuracy, with this difference approaching statistical significance (*p* = 0.08). Sensitivity analyses confirmed the robustness of the findings, and no publication bias was detected.

**Conclusion:**

Among individuals aged 20 and younger, acute caffeine ingestion produces a small yet statistically significant beneficial effect on cognitive performance. Specifically, moderate improvements were observed in psychomotor speed and attention, alongside smaller gains in executive function. No significant moderators influencing this ergogenic effect were identified in the present meta-analysis. Importantly, the conclusion that habitual caffeine intake exerts no moderating impact is derived from only seven studies and may be limited by insufficient statistical power. These findings offer tentative support for the judicious use of caffeine to boost cognition among young athletes and healthy adolescents, yet caution is warranted given the small number of small-sample studies.

**Systematic review registration:**

This systematic review has been registered in PROSPERO (International Prospective Register of Systematic Reviews) under registration number CRD420261342996.

## 1 Introduction

Caffeine (1,3,7-trimethylxanthine) is the most widely consumed psychoactive substance globally and is commonly used by athletes across all age groups due to its well-established ergogenic effects (1, 2). Since its removal from the World Anti-Doping Agency’s Prohibited List in 2004 (3), the potential benefits of caffeine for enhancing sports performance have garnered considerable attention (4–6). In the fast-paced context of competitive athletics, where millisecond-level reaction times, sustained attention, and precise decision-making often determine success (7, 8), athletes—particularly young athletes who are in critical periods of neurocognitive development—frequently consume moderate doses (approximately 3–6 mg/kg) of caffeine to achieve marginal gains in alertness, reaction speed, and executive function, thereby seeking a competitive edge in high-stakes environments (7, 9). However, the procognitive effects of caffeine are not uniform across individuals; their efficacy is highly dependent on individual differences (10, 11), with habitual daily caffeine consumption serving as one of the most critical moderating variables (12, 13).

Despite the widespread use of caffeine among young athletes and healthy individuals, findings from existing studies on its cognitive effects remain notably inconsistent. This inconsistency is particularly evident across different cognitive domains and dosage regimens. For example, in basic cognitive processes such as reaction time and attention, some studies report a beneficial effect only at a dose of 3 mg/kg, with higher doses (6 and 9 mg/kg) showing no improvement (14); conversely, other studies have observed cognitive enhancement at higher doses as well (15). Similarly, in higher-order cognitive functions such as executive control, evidence is mixed, with some studies showing no acute benefit (15, 16) and others reporting significant improvements following caffeine intake (17). These discrepancies likely stem from methodological heterogeneity across studies, including variations in administration routes (e.g., capsules, beverages), outcome measurement timing, and insufficient control for participants’ baseline fatigue and habitual caffeine consumption (18). Given these inconsistencies, a quantitative synthesis of the available evidence is warranted. To date, however, no meta-analysis has systematically quantified the effects of acute caffeine intake on cognitive performance specifically in populations aged 20 years and younger.

Therefore, a systematic synthesis and quantitative integration of the existing evidence is particularly warranted. Young caffeine consumers represent an ideal model for investigating the “true” procognitive effects of caffeine, as they exhibit higher sensitivity to caffeine and are less susceptible to withdrawal-related confounding (17, 19). Meta-analytic approaches allow for the quantification of overall effect sizes for specific cognitive domains—such as reaction time, attention, and executive function—and enable the exploration of dose–response relationships (18, 20, 21). Such an approach can not only help resolve the current inconsistencies in the literature but also provide precise, evidence-based recommendations regarding the efficacy, optimal dosage, and safety of caffeine use in this specific population.

Accordingly, this study aims to rigorously compare the effects of acute caffeine intake versus placebo on various cognitive functions in young caffeine consumers using systematic review and meta-analytic methods. We will focus on the effects of different doses on core cognitive domains, including reaction speed, attention, and executive function, with the goal of providing precise and safe caffeine use strategies for young populations.

## 2 Materials and methods

### 2.1 Study protocol

This systematic review and meta-analysis was conducted in accordance with the Preferred Reporting Items for Systematic Reviews and Meta-Analyses (PRISMA) guidelines (22) and was registered with the International Prospective Register of Systematic Reviews (PROSPERO) under the registration number CRD420261342996.

### 2.2 Literature search

Two authors (HL and JN) independently searched the Web of Science, PubMed, MEDLINE, and Cochrane Library databases from inception to March 18, 2026 (see Supplementary Table S1). The electronic database search employed a Boolean logic combination of keywords (“AND” “OR” and “NOT”): (“caffeine” OR “coffee” OR “caffeine capsules” OR “caffeinated gum” OR “caffeine mouth rinse” OR “caffeine pills” OR “caffeine drinks”) AND (“cognitive function” OR “cognition” OR “reaction time” OR “attention” OR “executive function” OR “working memory”) AND (“aged ≤ 20” OR “youth” OR “minors” OR “adolescents” OR “teenagers” OR “youth” OR “juveniles” OR “young people”) NOT (“review” OR “overview” OR “meta”). In addition, a secondary search strategy was employed involving manual screening of the reference lists of eligible studies (i.e., citation tracking). The search was restricted to English-language publications. Any discrepancies arising during this process were resolved through discussion between the two authors (HL and JN), with input from a third author (JL).

### 2.3 Selection criteria

Inclusion criteria were established according to the PICOS framework: (1) Population: healthy individuals under normal conditions, aged ≤20 years, without cognitive impairments, and not using any medications known to significantly affect cognitive function; (2) Intervention: acute caffeine administration via oral routes (including caffeine capsules, caffeinated gum, caffeine pills, and caffeine drinks, excluding caffeine mouth rinse); (3) Comparator: placebo control under identical conditions; (4) Outcome measures: cognitive function assessments, such as global cognition, executive function, attention, memory, and psychomotor speed; (5) Study design: randomized controlled trials or randomized crossover trials. Exclusion criteria were as follows: (1) lack of cognitive outcome measures or failure to report specific numerical values of means and standard deviations/errors for relevant outcomes; (2) review articles or conference abstracts; (3) duplicate publications; (4) non-English publications.

The reason for excluding studies using caffeine mouth rinsing was that the exact dosage of caffeine intake could not be accurately quantified.

### 2.4 Data extraction

Data extraction was conducted independently by two authors (HL and JN) in accordance with the Cochrane Handbook for Systematic Reviews of Interventions. Extracted information included study characteristics (authors and year), participant details (age, sex, and caffeine consumption habits), sample size, intervention parameters (administration method, dosage, washout period, and post-administration waiting time), and cognitive outcome measures (e.g., global cognition, executive function, attention, memory, and psychomotor speed). For studies reporting multiple cognitive domain tests, we applied a hierarchical selection procedure: the primary outcome was extracted if prespecified by the original study; otherwise, tests assessing a single cognitive construct (e.g., executive function, working memory, attention) were prioritized over composite scores. For studies examining multiple doses, we selected the dose recommended by the original authors or the clinically equivalent dose; when no preference was indicated, we chose the dose most representative of real-world use (e.g., a common daily dose rather than an excessively high loading dose).

For outcome measures with discrepancies between the two authors, a third author (Jia Li) was consulted to reach consensus through discussion. For each study, the mean and standard deviation of cognitive outcomes post-intervention were extracted. When a study reported multiple results for the same cognitive test, only a single outcome measure was extracted to avoid bias from duplication. For studies reporting results as means and standard errors, the following formula was applied:

$$\mathrm{SD}(\mathrm{pre})=\mathrm{SE}(\mathrm{pre})\sqrt{n};\mathrm{SD}(\text{post})=\mathrm{SE}(\text{post})\sqrt{n}$$

When data were presented exclusively in graphical format, WebPlotDigitizer (Ankit Rohatgi, 2019, version 4.2; WebPlotDigitizer, Pacifica, CA, USA) was used to extract the data from the figures. In cases where relevant data were unavailable, the corresponding author (Jia Li) contacted the first author or corresponding author of the original study via email to request the necessary data.

### 2.5 Quality assessment

The quality of the included studies was independently assessed by two authors (HL and JN) using the Physiotherapy Evidence Database (PEDro) scale. In cases where consensus could not be reached, a third author (JL) was consulted to resolve discrepancies through discussion. The PEDro scale evaluates key methodological elements, including randomization, blinding, and allocation concealment, which are essential for ensuring the internal validity of the studies included in this systematic review and meta-analysis (23). The PEDro scale comprises 11 items, each scored as 0 or 1. A score of 1 indicates that the criterion is met, whereas a score of 0 indicates non-compliance. Item 1 is not included in the total score; thus, the maximum possible score for the remaining 10 items (items 2 to 11) is 10. Studies with a methodological quality score of ≥9 were classified as excellent, those with scores of 6–8 as good, those with scores of 4–5 as fair, and those with scores of <4 as poor.

The quality of evidence was independently assessed by two authors (HL and JN) based on the Grading of Recommendations Assessment, Development and Evaluation (GRADE) framework. This grading system evaluates evidence across several domains, including study limitations, imprecision, inconsistency, indirectness, and publication bias. In the event of disagreement between the two authors on a given rating, the final author (JL) was consulted, and discussion continued until consensus was reached.

### 2.6 Statistical analyses

Given the inclusion of multiple cognitive outcomes across the included studies, the comparative fit of a three-level meta-analytic model versus a two-level model was further evaluated. The results indicated that the three-level model did not significantly improve model fit compared with the two-level model, in which the third level of heterogeneity was constrained to zero (LRT = 1.07, *p* = 0.3, see Supplementary Table S2). Therefore, the traditional two-level meta-analytic model was ultimately adopted over the more complex three-level alternative. This decision was based on the consideration that a three-level model is only practically valuable when it better captures the structural variability of the data—whereas, in this case, the goodness-of-fit was comparable between the two approaches. In accordance with Occam’s razor, the more parsimonious two-level model adequately accounted for the data variability while substantially reducing model complexity (24). Notably, given the heterogeneity across cognitive tasks, our pre-specified primary analysis consisted of subgroup analyses, with the overall pooled analysis serving only as a preliminary overview.

Statistical analyses were performed using R version 4.5.0 (R Foundation for Statistical Computing) with the meta, metafor, and dplyr packages (25–27). For crossover trials, we calculated standardized mean differences using formulas that incorporate the within-participant correlation between treatment periods (24, 28, 29). A correlation coefficient of 0.5 was assumed for the primary analysis, with sensitivity analyses using values of 0.3 and 0.7 to test the robustness of the findings. Given the inherent heterogeneity across the included studies—particularly in terms of experimental paradigms, procedural details, and potential moderating factors—we elected to use a random-effects model for all primary analyses, as it provides a more conservative and generalizable estimate of the overall effect. Meta-analytic results were presented as forest plots. Effect sizes were interpreted as trivial (Hedges’ *g* < 0.2), small (0.2 ≤ Hedges’ *g* < 0.5), moderate (0.5 ≤ Hedges’ *g* < 0.8), or large (Hedges’ *g* ≥ 0.8) (30). Between-study heterogeneity was quantified using the *I*^2^ statistic, with the between-study variance τ^2^ reported concurrently. *I*^2^ values of 0–25% were categorized as low heterogeneity, 25–50% as moderate heterogeneity, and > 50% as substantial heterogeneity (31). The threshold for statistical significance was set at *p* < 0.05.

### 2.7 Data robustness

To comprehensively assess the robustness of the pooled effect sizes across all meta-analytic comparisons, we conducted a series of sensitivity analyses. First, a leave-one-out analysis was performed using the metacont function from the R package meta to examine whether any individual study or effect size unduly influenced the overall consistency of the results. Second, we fitted a three-level meta-analytic model using the metafor package in R to account for potential non-independence among effect sizes. Additionally, we repeated the primary analyses using assumed correlation coefficients of 0.3 and 0.7, respectively, to evaluate the sensitivity of the results to varying levels of dependence.

To identify potential risks of publication bias, funnel plot visual inspection and Egger’s regression test were performed on the effect size data. When evidence of publication bias was present, the Duval and Tweedie trim-and-fill method was applied to adjust the pooled effect estimate at risk of bias, thereby estimating the true effect size (32).

### 2.8 Moderator effects

Nine moderating factors were identified in this study: age, sex (male, female and unclear), population (athletes and general population), administration method (capsule and liquid), intervention dose, habitual dose, intervention-to-habitual dose ratio (intervention dose / habitual dose), cognitive function type (psychomotor speed, attention, and executive function), and outcome data type (reaction time and accuracy). Age, habitual dose, and intervention-to-habitual dose ratio were continuous variables and were examined using meta-regression. Sex, population, administration method, intervention dose, cognitive function type, and data type were categorical variables and were examined using subgroup analysis.

## 3 Results

### 3.1 Study selection

The initial search yielded 2,098 records. After 754 duplicates were removed, 1,344 records advanced to title/abstract screening, from which 1,273 were excluded. The remaining 71 full-text articles were assessed for eligibility; 59 of these were excluded, leaving 12 studies. An additional two records were identified through citation searching, resulting in a total of 14 studies included in the final analysis (7, 8, 14–17, 21, 33–39)(Figure 1).

**Figure 1:**
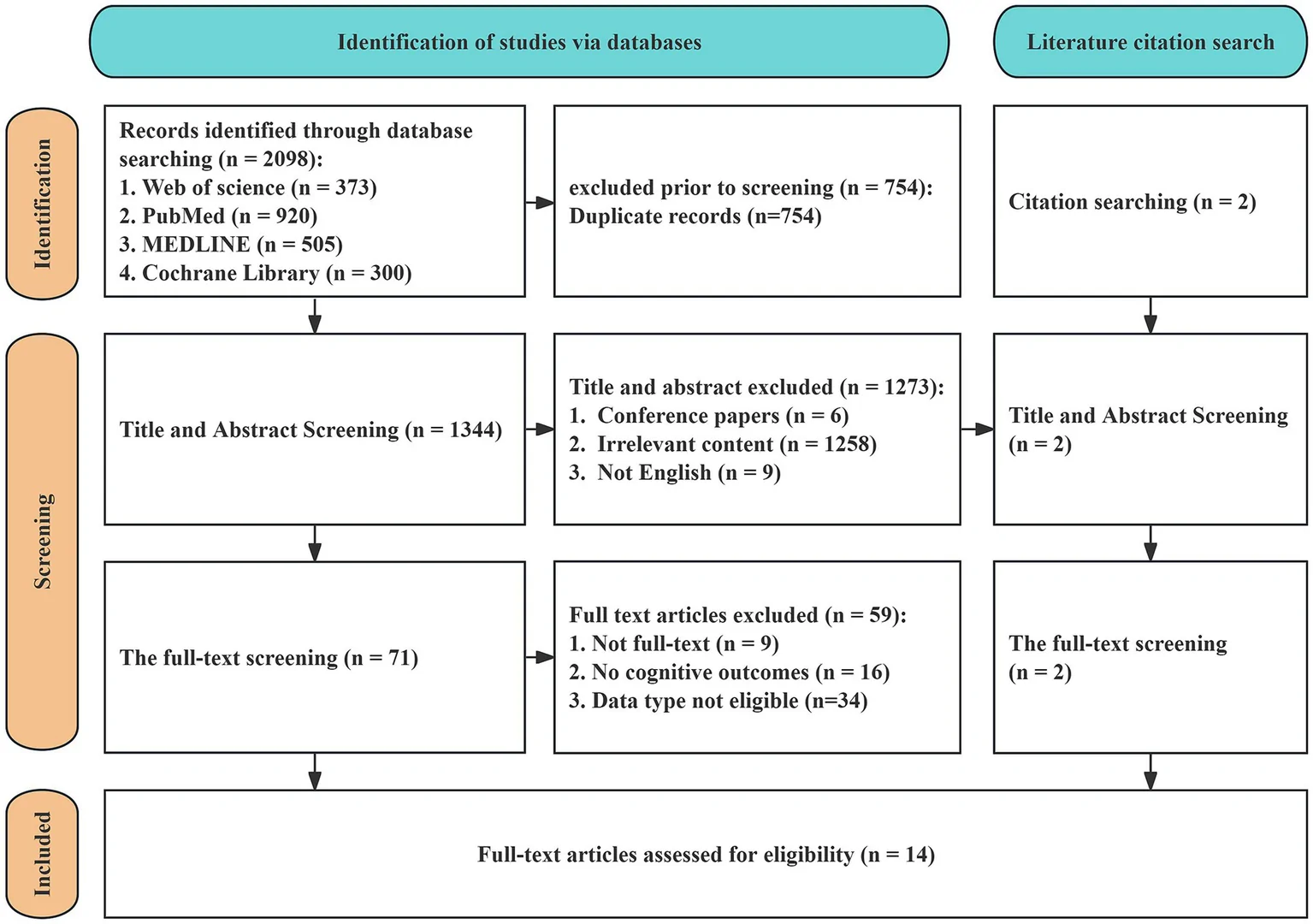
Flow diagram of the study selection process.

### 3.2 Study characteristics

This meta-analysis included data from 250 participants, comprising 143 males and 107 females. Among them, 124 were professional athletes, 112 were ordinary people, and 14 were active-duty military personnel. The mean age was 17.9 years (age range: 10.6 to 20 years). The studies were conducted in China (*n* = 3), Australia (*n* = 1), Tunisia (*n* = 4; one of which also included Qatar), the United Kingdom (*n* = 2), the United States (*n* = 1), Colombia (*n* = 1), Iran (*n* = 1), and Thailand (*n* = 1). Regarding study design, 13 were randomized controlled crossover trials (7, 8, 14, 15, 17, 21, 33–39) and one was a randomized controlled trial (16). Regarding outcome measures, this meta-analysis synthesized 37 effect sizes from 14 studies, covering multiple domains of cognitive function, including psychomotor speed (7, 8, 15, 37, 38), attention (15, 21, 33, 37–39), executive function (7, 8, 14–17, 35, 36, 38), and memory (21). Outcome measures included cognitive test reaction time (*k* = 20) and accuracy (*k* = 16). Detailed characteristics are presented in Table 1.

**Table 1:** Characteristics of included studies.

References	Study design	Sample size (M/F)	Age	Population	Withdrawal duration (hour)	post-administration waiting time (min)	Intervention dose	Administration route	Outcome measure
Amor et al. (8)	RCT crossover	*N* = 24 (24/0)	16.9	athletes	24	30	3 mg/kg	Capsule	SRT (RT) ↑; CRT (RT) →; Stroop Test (RT) ↑; Stroop Test (AC) ↑
Barry et al. (17)	RCT crossover	*N* = 24 12/12	10.6	ordinary people	4	38.1	NR	Capsule	Go/No Go (RT) ↑; Go/No Go (AC) ↑
Bougrine et al. (39)	RCT crossover	*N* = 15 (0/15)	16.3	athletes	24	60	6 mg/kg	Capsule	SRT (RT) ↑; Attention Test (AC) ↑
Bougrine et al. (15)	RCT crossover	*N* = 14 (0/14)	17.4	athletes	24	60	6 mg/kg	Capsule	SRT (RT) ↑; CRT (RT) ↑; Attention Test (AC) ↑; MRT (RT) ↑; MRT (AC)→
Duncan et al. (35)	RCT crossover	*N* = 13 (6/7)	20	ordinary people	48	60	3 mg/kg	Liquid	CAT (AC) ↑
Feng et al. (7)	RCT crossover	*N* = 15 (15/0)	20	athletes	24	45	5 mg/kg	tablet	Anticipation Accuracy (AC) ↑; SRT (RT) ↑
Jordan et al. (34)	RCT crossover	*N* = 17 (17/0)	14.1	athletes	24	60	6 mg/kg	Capsule	Dominant Side (RT) →
Kammerer et al. (21)	RCT crossover	*N* = 14 (14/0)	20	soldier	5	45	1.18 mg/kg	Liquid	FAT (AC) →; DS (AC) →
Nana et al. (33)	RCT crossover	*N* = 13 (13/0)	20	athletes	12	60	3.4 mg/kg	Capsule	AOP (RT) →; AOP (AC) →
Razazan et al. (36)	RCT crossover	*N* = 12 (12/0)	17	athletes	48	60	3 mg/kg	Capsule	Stroop Test (RT) → ; Stroop Test (AC) →
Romdhani et al. (37)	RCT crossover	*N* = 14 (14/0)	19.8	athletes	NR	30	5 mg/kg	Capsule	SRT (RT) →; CRT (RT) →
Smith et al. (38)	RCT crossover	*N* = 25 (6/19)	19	ordinary people	15.5	25	2 mg/kg	Liquid	FAT (RT) →; CST (RT) ↑; SRT (RT) ↑; RDDT (AC) ↑
Yuan et al. (16)	RCT	*N* = 40 (0/40)	19.3	ordinary people	NR	65	3.6 mg/kg	Capsule	3-back task (AC) ↑; 3-back task (RT) →; Stroop Test (AC) →; Stroop Test (RT) →; MOST (AC) →; MOST (RT) →
Zhang et al. (14)	RCT crossover	*N* = 10 (10/0)	20	ordinary people	24	60	6 mg/kg	Capsule	Stroop Test (RT) ↑; Stroop Test (AC) →

AC, accuracy; AOP, auditory oddball paradigm; CAT, coincidence anticipation timing; CRT, choice reaction time; CST, categoric search task; DS, digit span WAIS subtest; FAT, focused attention task; MOST, more-odd shifting task; MRT, mental rotation test; NR, not reported; RCT, randomized controlled trial; RDDT, repeated digits detection task; RT, reaction time; SRT, simple reaction time. ↑, indicates significant improvement in the outcome measures; →, indicates no significant improvement.

### 3.3 Study quality

The mean methodological quality score across all studies was 9.07 ± 0.9 (see Supplementary Table S3). Of these, 11 studies were rated as excellent (score ≥ 9), and three as good (score 6–8). Among all quality assessment items, the lowest-scoring domain was concealed allocation, which was met by only six studies (42.86%). Overall, all studies demonstrated a high level of methodological rigor. Regression analysis examining the association between study quality score and effect size showed no significant correlation (estimate = −0.07, *p* = 0.51), suggesting that differences in study quality are unlikely to have introduced bias.

### 3.4 Meta-analysis

Meta-analysis results (comprising 37 effect sizes from 14 studies) showed that acute caffeine intake had a small positive effect on cognitive task performance compared with placebo (SMD = 0.41, 95% CI = 0.27 to 0.55, *p* < 0.01, prediction interval = 0.11 to 0.72, Figure 2), with heterogeneity assessed as low (*I*^2^ = 10%, τ^2^ = 0.02, *p* = 0.3). Meta-regression analyses indicated that age, acute intervention dose, habitual daily caffeine intake, and the ratio of acute to habitual dose did not significantly moderate caffeine’s beneficial effects on cognitive task performance (Table 2). Nevertheless, these findings are likely constrained by insufficient statistical power. Subgroup analyses revealed no significant moderation of caffeine’s cognitive effects by cognitive domain, participant population, administration route, or sex (Table 3). Notably, however, the magnitude of caffeine-induced improvements in reaction time was markedly larger than that for accuracy, with this difference approaching statistical significance (*p* = 0.08).

**Figure 2:**
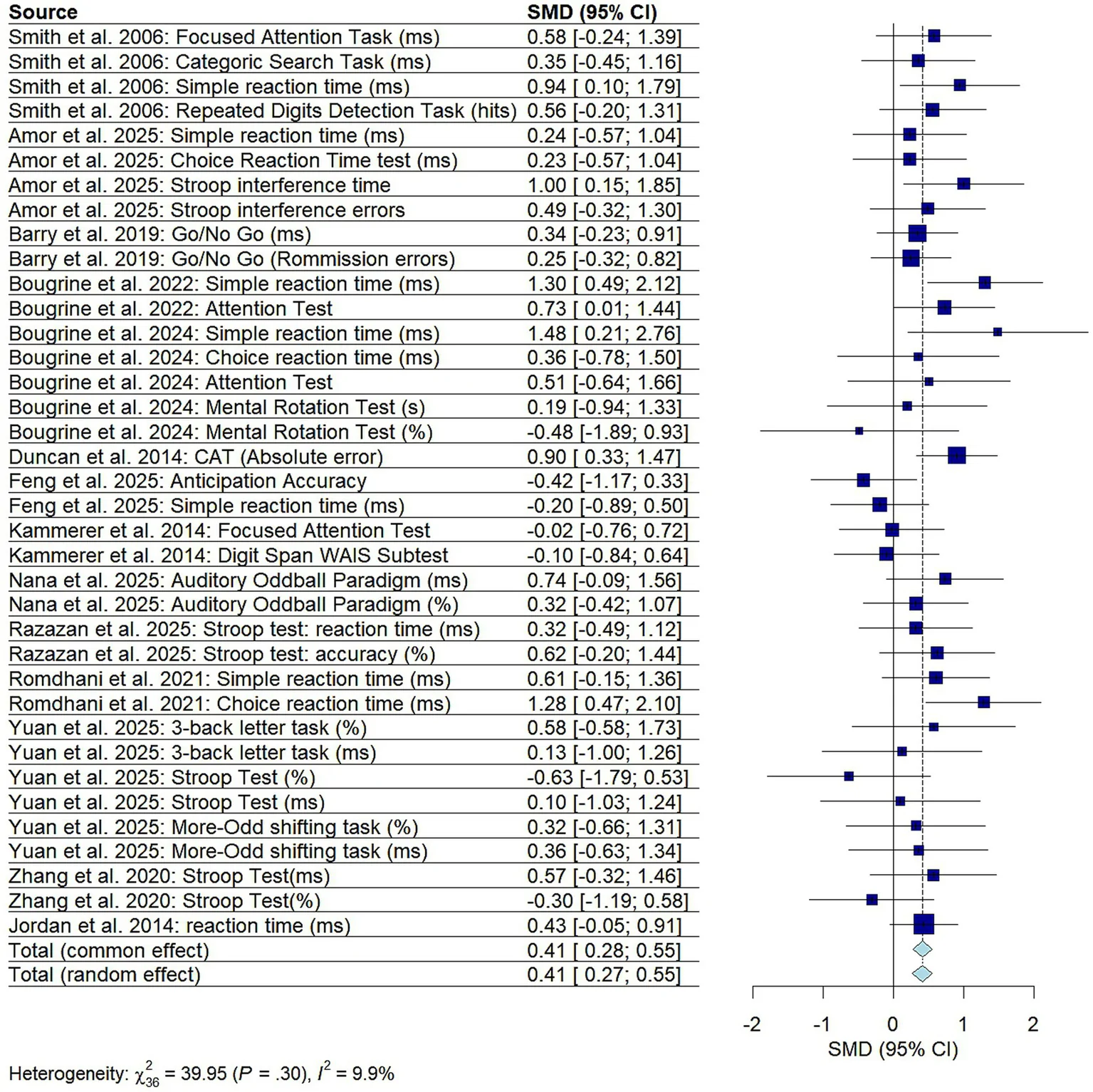
Forest plot of effect size for caffeine versus placebo.

**Table 2:** Meta-regression analysis of continuous variables.

Sub-group	*N*	*K*	Beta	*P*	Sig.	*τ* ^2^	*I* ^2^
Age	14	37	−0.0062	0.84	No	0	0
Intervention dose	13	35	0.0167	0.79	No	0.01	3
Daily dose	7	19	0.0002	0.93	No	0.01	6
Dose ratio	7	19	0.009	0.89	No	0.01	5

N, number of studies; K, number of effect sizes; Beta, regression coefficients for between-subgroup comparisons; P, significance value; τ2, measure of variation around average; I2, measure of proportion of observed variance (%).

**Table 3:** Subgroup analysis of categorical variables.

**Sub-group**	**N**	**K**	**SMD**	**95% CI**		**P**	**Sig.**	**τ** ^**2**^	**I** ^**2**^	**Beta**
Cognitive category										
Executive function	9	19	0.34	0.15	0.53	<0.01	Yes	0.02	0	ns
Psychomotor speed	6	9	0.5	0.24	0.75	<0.01	Yes	0.07	33	ns
Attention	6	8	0.55	0.26	0.83	<0.01	Yes	0	0	ref
Data type										
Completion time	12	21	0.55	0.38	0.72	<0.01	Yes	0.01	3	ns
Accuracy	11	16	0.21	0.002	0.42	<0.05	Yes	0	0	ref
Population										
Athletes	8	20	0.47	0.29	0.65	<0.01	Yes	0.05	24	ref
general	5	15	0.41	0.21	0.62	<0.01	Yes	0	0	ns
Administration route										
Capsule	10	28	0.46	0.30	0.62	<0.01	Yes	0	0	ns
Liquid	3	7	0.49	0.21	0.76	<0.01	Yes	0.05	21	ref
Gender										
Male	8	17	0.33	0.14	0.51	<0.01	Yes	0.04	22	ns
Female	3	13	0.47	0.19	0.76	<0.01	Yes	0.04	10	ns
Unclear	3	7	0.54	0.28	0.79	<0.01	Yes	0	0	ref

N, number of studies; K, number of effect sizes; SMD, standardized mean difference; 95% CI, lower and upper confidence intervals; P, significance value; τ2, measure of variation around average; I2, measure of proportion of observed variance (%); Beta, regression coefficients for between-subgroup comparisons; ref, reference category; ns, no significant difference compared to reference category.

### 3.5 Sensitivity analysis

To test the robustness of our primary findings, we performed sensitivity analyses using two different correlation coefficients (*r* = 0.3 and *r* = 0.7). The pooled effect sizes were virtually identical across models (*r* = 0.3: SMD = 0.36, 95% CI = 0.20 to 0.52, *p* < 0.01; *r* = 0.7: SMD = 0.50, 95% CI = 0.30 to 0.70, *p* < 0.01), confirming the robustness of our findings.

Sensitivity analyses based on the three-level meta-analytic model revealed a small but statistically significant beneficial effect of caffeine on cognitive task performance (SMD = 0.40, 95% CI = 0.21–0.59, *p* < 0.01, see Supplementary Figure S1). This pooled effect estimate was consistent with that derived from the two-level model, indicating the findings were robust to alternative analytical specifications.

Leave-one-out sensitivity analysis revealed that the effect remained significantly positive after the sequential removal of each study (number of effect sizes ranging from 33 to 36, SMD ranging from 0.38 to 0.47, 95% CI ranging from 0.23 to 0.6, *p* < 0.01, see Supplementary Table S4), with only minor changes in heterogeneity (*I*^2^ ranging from 0 to 17%). Collectively, these sensitivity analyses indicate that the results of this meta-analysis are robust.

### 3.6 Risk of bias and GRADE

Funnel plot symmetry test (Figure 3) and Egger’s test (intercept = −0.3, *t* = −0.4, *p* = 0.58) indicated a low likelihood of publication bias among the included studies, indicating the robustness of the meta-analytic findings. Regression analysis of effect size against sample size indicated a low likelihood of small-study bias across the included studies (estimate = −0.008, *p* = 0.3). Additionally, the overall quality of evidence for cognitive outcomes, based on the 14 included studies, was rated as moderate, having been downgraded by one level due to inconsistency across study findings (see Supplementary Table S5).

**Figure 3:**
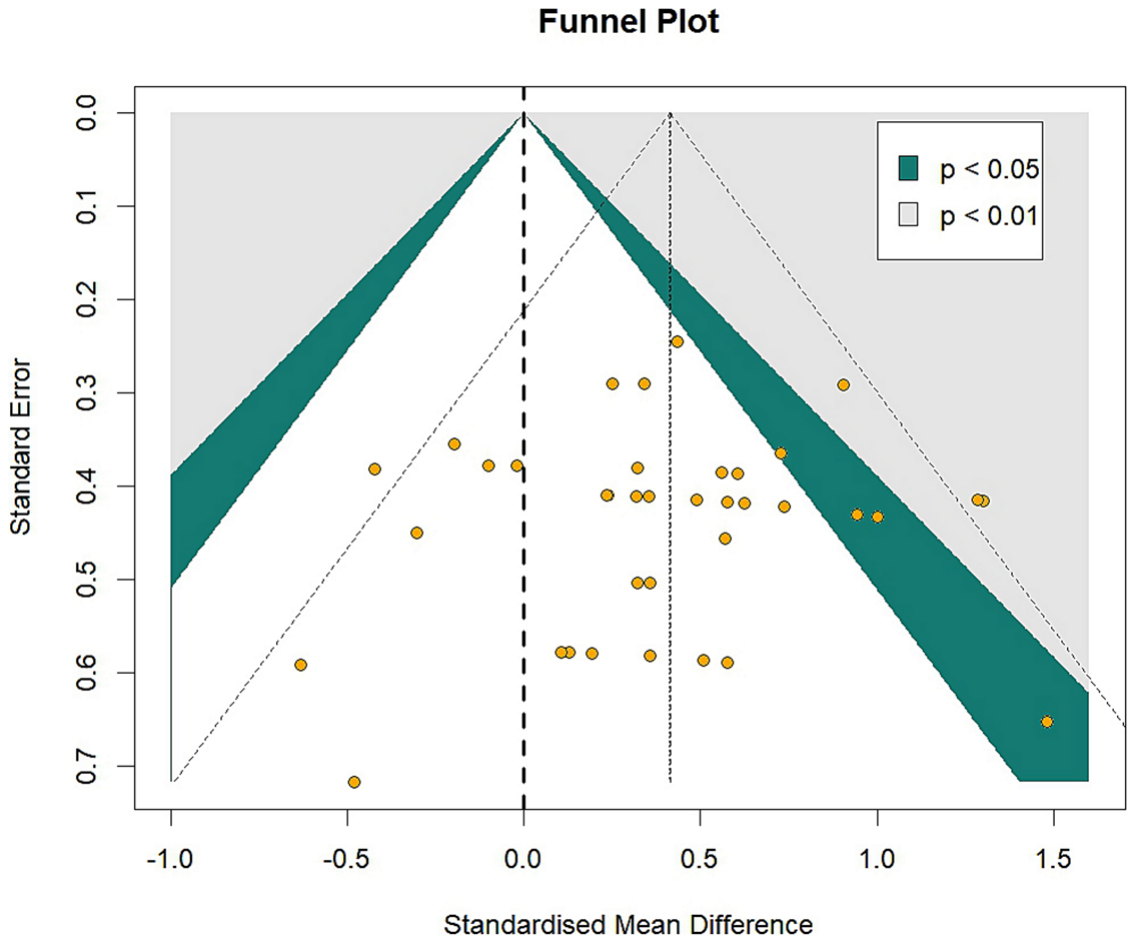
Contour-enhanced funnel plot of cognitive function.

## 4 Discussion

This meta-analysis is the first to quantify the effects of acute caffeine intake on cognitive function among young adults aged 20 years and below. Pooled data from 14 studies revealed modest improvements in cognitive task performance following caffeine consumption in this population (SMD = 0.41). Specifically, caffeine yielded moderate enhancements in attention (SMD = 0.55) and psychomotor speed (SMD = 0.5), yet produced only small improvements in executive function (SMD = 0.34). Meta-analyses of other cognitive domains were not feasible owing to an insufficient number of eligible studies. An important finding was that caffeine’s beneficial effects differed by outcome type: caffeine produced small gains for task accuracy (SMD = 0.21) and moderate gains for task speed (SMD = 0.55). Additionally, no evidence supported that age, population type, sex, acute intervention dosage, habitual daily caffeine intake, acute-to-habitual caffeine dose ratio, or administration route served as potential moderators of caffeine’s ergogenic effects.

Caffeine exerts its effects by blocking adenosine A₁ and A2a receptors, thereby relieving the inhibition of neuronal activity and enhancing cortical excitability and alertness (40). This mechanism directly optimizes the initial processing stages of stimulus perception and motor initiation, accounting for the most pronounced improvements in basic cognitive tasks such as reaction time and attention (41, 42). Consistent with this, multiple studies included in our analysis confirmed that caffeine significantly enhances performance on simple reaction time and sustained attention tasks (15, 38, 39). However, performance on tasks requiring higher-order cognitive control relies not only on psychomotor speed but also on the precise regulation of prefrontal cortical networks involved in inhibitory control and working memory updating (16, 43, 44). The indirect support provided by caffeine through non-specific arousal systems is insufficient to substantially optimize such complex cognitive processes, particularly in young, healthy populations in whom these functions are inherently at a high baseline level with limited capacity for improvement (45). This may partly explain the phenomenon of “neuro-behavioral dissociation” observed in some studies, whereby caffeine enhances P300 amplitude without necessarily translating into improved behavioral performance (33). Similarly, even when the neural component associated with inhibitory control, N2b, is enhanced, whether such neural-level improvements translate into behavioral benefits remains contingent on task difficulty and individual cognitive reserve (17, 36).

Although meta-regression analysis did not reveal a significant association between intervention dose and overall cognitive effect size (Beta = 0.005, *p* = 0.93), this null finding does not preclude the possibility of a dose–response relationship, particularly given the limited number of studies included in the meta-regression. A qualitative review of the primary studies does suggest a complex, non-linear pattern: for instance, one study showed cognitive benefits at 3 mg/kg and 6 mg/kg, but not at 9 mg/kg, with the higher dose being associated with the greatest adverse effects (14, 15). These observations are consistent with the pharmacological principle that low doses of caffeine may adequately antagonize adenosine receptors, while higher doses carry an increased risk of overstimulation (46). However, we acknowledge that the current evidence base is insufficient to define an optimal dose. The available data do not permit a robust recommendation of ~3 mg/kg as the superior dose, as the dose–response relationship was not statistically confirmed and safety outcomes were not systematically evaluated across studies. Instead, we suggest that future research should prioritize systematic, well-powered dose-finding studies that incorporate standardized safety assessments to determine whether a low-dose range (e.g., approximately 3 mg/kg) may offer a favorable balance between cognitive efficacy and tolerability in pediatric and adolescent populations.

Subgroup analyses revealed no significant moderation of caffeine’s cognitive effects by cognitive domain, participant population, administration route, or sex (Table 3). However, a notable pattern emerged when comparing cognitive outcome types: the magnitude of caffeine-induced improvements was greater for reaction time than for accuracy, with this difference approaching statistical significance (*p* = 0.08). This trend, while not reaching conventional significance, may suggest that caffeine affects processing speed more robustly than response precision. One plausible explanation is that caffeine’s primary mechanism—antagonism of central adenosine receptors—enhances vigilance and arousal, which are more directly captured by reaction time measures (37, 38, 40). Accuracy, in contrast, is influenced by a wider array of factors, including task difficulty and ceiling effects, which may obscure subtle drug-related enhancements (45). Given this observed trend, future studies should consider using task paradigms that are sufficiently sensitive to detect changes in both speed and accuracy, and should avoid relying on a single outcome measure when evaluating caffeine’s cognitive effects in pediatric and adolescent populations.

## 5 Limitations

When interpreting the findings of this study, several limitations should be considered. First, although the overall analysis indicated a low risk of publication bias, the included studies generally had small sample sizes (mean sample size = 17.9), which may result in insufficient statistical power, particularly in subgroup analyses and meta-regression, potentially precluding the detection of genuine moderating effects. Second, most studies assessed only the acute effects of caffeine, lacking follow-up on long-term use, tolerance, and withdrawal symptoms. Third, only seven of the included studies reported detailed information on participants’ habitual caffeine intake, while the remaining seven did not. This incomplete reporting may introduce potential confounding bias and weaken the quality of evidence regarding the relationship between daily consumption and effect size. Fourth, sex differences remain underexplored. Although subgroup analyses revealed no moderating effect of sex on the overall effect, the limited number of studies with female participants restricts the certainty of the evidence. Future large-scale studies specifically targeting female populations and accounting for menstrual cycle phase are warranted. Fifth, the study populations were highly heterogeneous, including elite athletes from various sports and non-athletic students. These groups differ fundamentally in baseline fitness, cognitive demands of their training/occupation, and potentially caffeine habituation, which could influence the observed effects. While our subgroup analysis did not find a significant moderating effect of population type, the lack of statistical power prevents definitive conclusions.

Future research should employ prospective designs with larger sample sizes to validate the effects of varying doses. Integrating neuroimaging techniques with genotyping may further elucidate the neural mechanisms underlying the effects of caffeine and the sources of interindividual variability. Additionally, there is a need to develop cognitive tasks with greater ecological validity that are tailored to sport-specific contexts to evaluate the effects of caffeine in real-world competitive settings.

## 6 Conclusion

Among individuals aged 20 and younger, acute caffeine ingestion produces a small yet statistically significant beneficial effect on cognitive performance. Specifically, moderate improvements were observed in psychomotor speed and attention, alongside smaller gains in executive function. No significant moderators influencing this ergogenic effect were identified in the present meta-analysis. Importantly, the conclusion that habitual caffeine intake exerts no moderating impact is derived from only seven studies and may be limited by insufficient statistical power. These findings offer tentative support for the judicious use of caffeine to boost cognition among young athletes and healthy adolescents, yet caution is warranted given the small number of small-sample studies.
