# Supplementary material for: Acute caffeine intervention and cognitive performance in populations aged 20 years and younger: a systematic review and meta-analysis
Source: Front Nutr. 2026 Sep 24;13:1888658. doi: 10.3389/fnut.2026.1888658 (PMC13645521; doi:10.3389/fnut.2026.1888658)
Supplement: Supplementary file 1 [file Supplementery_file_1.docx]

Supplementary Material

# 1 Supplementary Figures

**Supplementary Figure S1.** Forest plot of the three-level meta-analysis.

# 2 Supplementary Tables

**Table S1.** Search Sources and Corresponding Results.

| **Data source** | **Search query** | **Results** |
| --- | --- | --- |
| Web of science | ALL=(caffeine OR coffee OR caffeine capsules OR caffeinated gum OR caffeine pills OR caffeine drinks) AND ALL=(cognitive function OR cognition OR reaction time OR attention OR vigilance OR executive function OR working memory) AND ALL=(aged ≤ 20 OR youth OR minors OR adolescents OR teenagers OR youth OR juveniles OR young people) NOT TS=(review OR overview OR meta) | 373 |
| Pubmed | (((caffeine OR coffee OR caffeine capsules OR caffeinated gum OR caffeine pills OR caffeine drinks) AND (cognitive function OR cognition OR reaction time OR attention OR vigilance OR executive function OR working memory)) AND (aged ≤ 20 OR youth OR minors OR adolescents OR teenagers OR youth OR juveniles OR young people)) NOT (review OR overview OR meta) | 920 |
| Medline | (caffeine OR coffee OR caffeine capsules OR caffeinated gum OR caffeine pills OR caffeine drinks) AND (cognitive function OR cognition OR reaction time OR attention OR vigilance OR executive function OR working memory) AND (aged ≤ 20 OR youth OR minors OR adolescents OR teenagers OR youth OR juveniles OR young people) NOT (review OR overview OR meta) | 505 |
| Cochrane Library | (caffeine OR coffee OR caffeine capsules OR caffeinated gum OR caffeine pills OR caffeine drinks) AND (cognitive function OR cognition OR reaction time OR attention OR vigilance OR executive function OR working memory) AND (aged ≤ 20 OR youth OR minors OR adolescents OR teenagers OR youth OR juveniles OR young people) NOT (review OR overview OR meta) | 300 |

**Table S2.** Comparison of Model Fit Between Two-level and Three-level Meta-analysis Models.

| **Meta‑analytic model** | **df** | **AIC** | **BIC** | **AICc** | **logLik** | **QE** | **SMD** | **LRT** | **pval** |
| --- | --- | --- | --- | --- | --- | --- | --- | --- | --- |
| Full | 3 | 55.0825 | 59.8331 | 55.8325 | -24.5413 | 59.2817 | 0.4 |  |  |
| Reduced | 2 | 53.0825 | 56.2496 | 53.4462 | -24.5413 | 59.2817 | 0.41 | 1.0691 | 0.3011 |

Df: degrees of freedom; AIC: Akaike Information Criterion; BIC: Bayesian Information Criterion; AICc: corrected Akaike Information Criterion; logLik: log-likelihood; QE: Q-statistic for heterogeneity; SMD: standardized mean difference; LRT: likelihood ratio test; pval: p-value.

**Table S3.** Quality Assessment of Included Studies.

| **Study** | **Eligibility criteria** | **Random allocation** | **Concealed allocation** | **Similar baseline** | **Participant blinding** | **Investigator blinding** | **Assessor blinding** | **Completeness of follow-up** | **Intention to treat** | **Between group comparisons** | **Point measures and variability** | **Total Score** | **Overall quality** |
| --- | --- | --- | --- | --- | --- | --- | --- | --- | --- | --- | --- | --- | --- |
| Amor et al. 2025 | 1 | 1 | 1 | 1 | 1 | 1 | 1 | 1 | 1 | 1 | 1 | 10 | Excellent |
| Barry et al. 2019 | 1 | 1 | 0 | 1 | 1 | 1 | 1 | 1 | 1 | 1 | 1 | 9 | Excellent |
| Bougrine et al. 2022 | 1 | 1 | 0 | 1 | 1 | 1 | 1 | 1 | 0 | 1 | 1 | 8 | Good |
| Bougrine et al. 2024 | 1 | 1 | 1 | 1 | 1 | 1 | 1 | 1 | 1 | 1 | 1 | 10 | Excellent |
| Duncan et al. 2014 | 1 | 1 | 0 | 1 | 1 | 1 | 1 | 1 | 1 | 1 | 1 | 9 | Excellent |
| Feng et al. 2025 | 1 | 1 | 1 | 1 | 1 | 1 | 1 | 1 | 1 | 1 | 1 | 10 | Excellent |
| Jordan et al. 2014 | 1 | 1 | 0 | 1 | 1 | 1 | 0 | 1 | 1 | 1 | 1 | 8 | Good |
| Kammerer et al. 2014 | 1 | 1 | 1 | 1 | 1 | 1 | 1 | 1 | 1 | 1 | 1 | 10 | Excellent |
| Nana et al. 2025 | 1 | 1 | 1 | 1 | 1 | 1 | 1 | 1 | 0 | 1 | 1 | 9 | Excellent |
| Razazan et al. 2025 | 1 | 1 | 1 | 1 | 1 | 1 | 1 | 1 | 1 | 1 | 1 | 10 | Excellent |
| Romdhani et al. 2021 | 1 | 1 | 0 | 1 | 1 | 1 | 1 | 1 | 1 | 1 | 1 | 9 | Excellent |
| Smith et al. 2006 | 1 | 1 | 0 | 1 | 1 | 1 | 1 | 1 | 1 | 1 | 1 | 9 | Excellent |
| Yuan et al. 2025 | 1 | 1 | 0 | 1 | 1 | 0 | 0 | 1 | 1 | 1 | 1 | 7 | Good |
| Zhang et al. 2020 | 1 | 1 | 0 | 1 | 1 | 1 | 1 | 1 | 1 | 1 | 1 | 9 | Excellent |
| Total | 13 | 13 | 6 | 13 | 13 | 12 | 12 | 13 | 12 | 13 | 13 | 9.07 | Excellent |

**Table S4.** Subgroup Analysis of Categorical Variables.

| **Excluded studies** | **K** | **SMD** | **95% CI** | | **P** | **Sig.** | **τ^2^** | **I^2^** |
| --- | --- | --- | --- | --- | --- | --- | --- | --- |
| Amor et al. 2025 | 33 | 0.4 | 0.25 | 0.56 | <0.01 | Yes | 0.03 | 15.1 |
| Barry et al. 2019 | 35 | 0.43 | 0.27 | 0.58 | <0.01 | Yes | 0.03 | 14 |
| Bougrine et al. 2022 | 35 | 0.38 | 0.24 | 0.52 | <0.01 | Yes | 0.01 | 1 |
| Bougrine et al. 2024 | 32 | 0.41 | 0.27 | 0.56 | <0.01 | Yes | 0.02 | 13 |
| Duncan et al. 2014 | 36 | 0.39 | 0.25 | 0.52 | <0.01 | Yes | 0.001 | 5 |
| Feng et al. 2025 | 35 | 0.47 | 0.33 | 0.6 | <0.01 | Yes | 0 | 0 |
| Jordan et al. 2014 | 36 | 0.41 | 0.26 | 0.56 | <0.01 | Yes | 0.3 | 12 |
| Kammerer et al. 2014 | 35 | 0.45 | 0.3 | 0.59 | <0.01 | Yes | 0.01 | 7 |
| Nana et al. 2025 | 35 | 0.41 | 0.26 | 0.56 | <0.01 | Yes | 0.03 | 14 |
| Razazan et al. 2025 | 35 | 0.41 | 0.26 | 0.56 | <0.01 | Yes | 0.03 | 14 |
| Romdhani et al. 2021 | 35 | 0.38 | 0.24 | 0.52 | <0.01 | Yes | 0.01 | 3 |
| Smith et al. 2006 | 33 | 0.39 | 0.23 | 0.54 | <0.01 | Yes | 0.03 | 15.8 |
| Yuan et al. 2025 | 31 | 0.44 | 0.29 | 0.59 | <0.01 | Yes | 0.03 | 17 |
| Zhang et al. 2020 | 35 | 0.43 | 0.28 | 0.57 | <0.01 | Yes | 0.02 | 9 |

**Table S5.** Quality Assessment Form for Studies Included in the Movement Meta-Analysis.

| Certainty assessment | | | | | | | No of patients | | Effect | | Certainty | Importance |
| --- | --- | --- | --- | --- | --- | --- | --- | --- | --- | --- | --- | --- |
| No of studies | Study design | Risk of bias | Inconsistency | Indirectness | Imprecision | Other considerations | Caffeine | Placebo | Relative (95% CI) | Absolute (95% CI) |  |  |
| Caffeine vs Placebo | | | | | | | | | | | | |
| 14 | randomised trials | not serious | serious | not serious | not serious | none | 230 | 230 | - | SMD 0.41 SD higher (0.28 higher to 0.55 higher) | ⨁⨁⨁◯ Moderate | Critical |

CI: confidence interval; SMD: standardised mean difference; SD: standard deviation.
